# Identification, Expression Analysis, and Target Prediction of Flax Genotroph MicroRNAs Under Normal and Nutrient Stress Conditions

**DOI:** 10.3389/fpls.2016.00399

**Published:** 2016-04-06

**Authors:** Nataliya V. Melnikova, Alexey A. Dmitriev, Maxim S. Belenikin, Nadezhda V. Koroban, Anna S. Speranskaya, Anastasia A. Krinitsina, George S. Krasnov, Valentina A. Lakunina, Anastasiya V. Snezhkina, Asiya F. Sadritdinova, Natalya V. Kishlyan, Tatiana A. Rozhmina, Kseniya M. Klimina, Alexandra V. Amosova, Alexander V. Zelenin, Olga V. Muravenko, Nadezhda L. Bolsheva, Anna V. Kudryavtseva

**Affiliations:** ^1^Laboratory of Post-Genomic Research, Engelhardt Institute of Molecular Biology, Russian Academy of SciencesMoscow, Russia; ^2^Laboratory of Structural and Functional Genomics, Engelhardt Institute of Molecular Biology, Russian Academy of SciencesMoscow, Russia; ^3^Department of Higher Plants, Lomonosov Moscow State UniversityMoscow, Russia; ^4^Laboratory of Genetics, All-Russian Research Institute for FlaxTorzhok, Russia; ^5^Laboratory of Genetics of Microorganisms, Vavilov Institute of General Genetics, Russian Academy of SciencesMoscow, Russia; ^6^Laboratory of Molecular Karyology, Engelhardt Institute of Molecular Biology, Russian Academy of SciencesMoscow, Russia

**Keywords:** *Linum usitatissimum*, genotroph, nutrient stress, microRNA, gene expression, qPCR, reference gene, miR399

## Abstract

Cultivated flax (*Linum usitatissimum* L.) is an important plant valuable for industry. Some flax lines can undergo heritable phenotypic and genotypic changes (LIS-1 insertion being the most common) in response to nutrient stress and are called plastic lines. Offspring of plastic lines, which stably inherit the changes, are called genotrophs. MicroRNAs (miRNAs) are involved in a crucial regulatory mechanism of gene expression. They have previously been assumed to take part in nutrient stress response and can, therefore, participate in genotroph formation. In the present study, we performed high-throughput sequencing of small RNAs (sRNAs) extracted from flax plants grown under normal, phosphate deficient and nutrient excess conditions to identify miRNAs and evaluate their expression. Our analysis revealed expression of 96 conserved miRNAs from 21 families in flax. Moreover, 475 novel potential miRNAs were identified for the first time, and their targets were predicted. However, none of the identified miRNAs were transcribed from LIS-1. Expression of seven miRNAs (miR168, miR169, miR395, miR398, miR399, miR408, and lus-miR-N1) with up- or down-regulation under nutrient stress (on the basis of high-throughput sequencing data) was evaluated on extended sampling using qPCR. Reference gene search identified *ETIF3H* and *ETIF3E* genes as most suitable for this purpose. Down-regulation of novel potential lus-miR-N1 and up-regulation of conserved miR399 were revealed under the phosphate deficient conditions. In addition, the negative correlation of expression of lus-miR-N1 and its predicted target, ubiquitin-activating enzyme E1 gene, as well as, miR399 and its predicted target, ubiquitin-conjugating enzyme E2 gene, was observed. Thus, in our study, miRNAs expressed in flax plastic lines and genotrophs were identified and their expression and expression of their targets was evaluated using high-throughput sequencing and qPCR for the first time. These data provide new insights into nutrient stress response regulation in plastic flax cultivars.

## Introduction

Cultivated flax (*Linum usitatissimum* L.) is an important plant widely used in the textile industry and for production of a number of food, chemical, and pharmaceutical *products* (Cullis, 2007). Some flax lines (called plastic or Pl) can undergo phenotypic and genotypic changes in response to the specific nutrient conditions. These alterations arising in a plant can be transmitted to subsequent generations; such lines inheriting the alterations are termed genotrophs (Durrant, 1958, 1962; Evans et al., 1966; Cullis and Charlton, 1981; Tyson and Fieldes, 1982; Chen et al., 2005, 2009; Bickel et al., 2012). Two types of genotrophs are distinguished: the large (L) genotrophs, which are formed under excess nutrient, and the small (S) genotrophs, which are formed under nutrient deficiency (Durrant, 1962). L and S genotrophs differ from each other and from their parental Pl plants in phenotype, total DNA content, and copy number variation of ribosomal RNA genes (Durrant, 1958, 1962; Evans et al., 1966; Cullis and Charlton, 1981; Tyson and Fieldes, 1982; Chen et al., 2005, 2009; Bickel et al., 2012). Moreover, only S genotrophs are characterized by the presence of Linum Insertion Sequence 1 (LIS-1), this genome alteration is not observed in L genotrophs or parental Pl plants. LIS-1 is a 5.7-kb sequence that occurred into a specific target site of the flax genome, the function of which is still unknown (Chen et al., 2005, 2009; Bickel et al., 2012). Although flax genotrophs have been extensively studied, the mechanisms of their genetic and epigenetic regulation remain unclear (Cullis, 2005). It is assumed that microRNAs (miRNAs) are involved in the adaptation of flax plants to nutrient stress and in the process of genotroph formation. It has been suggested that LIS-1 encodes miRNAs that mediate the regulation of gene expression under nutrient deficiency (Bickel et al., 2012). For understanding of gene expression regulation in flax under stress condition, more information about miRNAs is needed.

MiRNAs are small RNAs (sRNAs) that negatively regulate gene expression by specifically binding to and cleaving their target mRNAs or by inhibiting their translation. They play an essential role in regulating many processes occurring in plants (Jones-Rhoades et al., 2006). Changes in the expression of certain miRNAs have been reported under various environmental stresses such as drought, salinity, cold, heat, hypoxia, pathogen infection, heavy metal, and high or low levels of nutrient elements (Sunkar et al., 2007; Guleria et al., 2011; Khraiwesh et al., 2012). However, miRNA sequences as well as information about the alterations in their expression are available only for a few plant species. To date, only computational data concerning prediction of novel flax miRNAs are available. Limited experimental data were obtained on differential expression of flax miRNAs under different stress conditions (Bickel et al., 2012; Moss and Cullis, 2012; Neutelings et al., 2012; Barvkar et al., 2013; Melnikova et al., 2014a, 2015).

In the present work, we continue our line of investigations devoted to miRNA expression analysis in flax plants under nutrient stress (Melnikova et al., 2014a, 2015). Here we represent the new results concerning identification of novel potential miRNAs using the high-throughput sequencing, search for LIS-1-derived miRNAs, selection of reference genes and miRNAs for qPCR expression analysis, prediction of miRNA targets, and evaluation of the expression level of miRNAs and their predicted targets on the extended sampling using qPCR. These data provide new insights into nutrient stress response regulation in plastic flax cultivars.

## Materials and methods

### Plant material

*L. usitatissimum* cultivars “Stormont Cirrus”, “TOST”, “Synichka”, and “Antey” were planted in 5″ pots with pearlite and grown in a climate chamber under a 16-h light/8-h dark cycle at 22°C for 6 weeks. The plants were grown under one of the following nutrition conditions: normal nutrition (N): 100 ml of 0.5 × Hoagland's solution (Hoagland and Arnon, 1950), pH 5.5, was applied weekly in each pot; excess nutrition (NPK): 100 ml of 0.5 × Hoagland's solution, pH 5.5, supplemented with 2.56 g/l MASTER (Valagro, Italy; N:P:K:: 13:40:13, and micronutrients—Mg, S, B, Fe, Mn, Zn, Cu, Mo) was applied every 7 days in each pot; inorganic phosphate deficient nutrition (P): 100 ml of 0.5 × Hoagland's solution with a hundredfold reduced KH_2_PO_4_, pH 5.5, was applied every 7 days in each pot; potassium concentration was compensated through potassium acetate buffer (S1 Table). The NPK and P conditions were previously reported to cause heritable changes in “Stormont Cirrus” (Durrant, 1958, 1962; Cullis and Charlton, 1981). Leaf samples were collected from individual flax plants after 6 weeks of growth and were immediately frozen in liquid nitrogen. Plant samples were stored at −70°C. Plant material was obtained from 15 plants of “TOST” (five plants were grown under N conditions, five—under P, and five—under NPK), six plants of “Antey” (3—N, 3—P), seven plants of “Synichka” (4—N, 2—P, 1—NPK), and five plants of “Stormont Cirrus” (N—2, P—2, NPK—1).

### LIS-1 detection

The LIS-1 status of each individual plant was tested using a sample of DNA isolated from upper leaves of 15 plants of “TOST”, six plants of “Antey”, seven plants of “Synichka”, and five plants of “Stormont Cirrus” after 6 weeks of growth. DNA was extracted as described previously (Melnikova et al., 2014b); chloroform:isoamyl alcohol (24:1) extraction; and ethanol precipitation were used. The PCR reactions were performed in a final volume of 25 μl, containing 1 × PCR mixture 5-Red (AmplySens, Russia), 3 mM of each dNTPs, 10 μM of forward and reverse primers and 20 ng of DNA. Primers 5′-GGGTTTCAGAACTGTAACGAA-3′ (forward) and 5′-GAGGATGGAAGATGAAGAAGG-3′ (reverse) for detecting the presence of LIS-1, 5′-GGGTTTCAGAACTGTAACGAA-3′ (forward), and 5′-GCTTGGATTTAGACTTGGCAAC-3′ (reverse) for detecting the absence of LIS-1 (Chen et al., 2009) were used. The results were assessed using agarose gel electrophoresis.

### RNA isolation

Total RNA was extracted from the frozen flax leaves using RNA MicroPrep kit (Zymo Research, USA) in compliance with the requirements of manufacturer. RNA quality and concentration were determined by NanoDrop ND-1000 spectrophotometer (NanoDrop Technologies Inc., USA) and Qubit 2.0 fluorometer (Life Technologies, USA). Only the samples with an A260/A280 ratio nearly 2.0 were used for further analysis.

### Small RNA sequencing and bioinformatics analysis

RNA quality was evaluated using Agilent 2100 Bioanalyzer (Agilent Technologies, USA). Only high-quality RNA samples with RNA Integrity Number (RIN) value not less than 8.0 were used for sRNA library preparation using Illumina TruSeq small RNA preparation kit (Illumina, USA) in compliance with manufacturer's sample preparation guide. For library preparation, three RNA samples of “Stormont Cirrus” plants grown under N, P, and NPK conditions were used. Purified sRNA library concentration was evaluated using Qubit 2.0 fluorometer. Three cDNA libraries were used for cluster generation on Illumina c-Bot and sequenced on Illumina GAIIx.

Raw sequence reads were cleaned by removing low-quality reads and adapter reads. For the next processing, we used cleaned reads with abundance six or more. The remaining high-quality reads were used to analyze length distribution and were mapped to the *L. usitatissimum* genome (Wang et al., 2012) using Bowtie mapping software (Langmead et al., 2009).

To identify known conserved miRNA in flax, sRNA sequences were aligned with known matured miRNA sequences from miRBase 19.0 (http://www.mirbase.org/, Kozomara and Griffiths-Jones, 2011) and PMRD (plant microRNA database, http://bioinformatics.cau.edu.cn/PMRD/). For filtering the read dataset from known non-miRNA sRNA sequences, we used the Rfam (http://rfam.sanger.ac.uk/) database. We then used miRCat (Stocks et al., 2012) to predict mature miRNAs and their precursors from a sRNA dataset and a flax genome (Wang et al., 2012).

Among the sRNA sequences that could not be annotated, novel potential miRNAs were identified using the mfold webserver (http://mfold.rna.albany.edu/?qjmfold/) that folds flanking sequences and predicts secondary structures of single stranded nucleic acids (Zuker, 2003). Target predictions were performed using psRNATarget (http://plantgrn.noble.org/psRNATarget/; Moxon et al., 2008; Dai and Zhao, 2011) with default parameters.

### Identification of LIS-1-derived miRNAs

After trimming the adapters, the derived miRNA candidate sequences (with abundance 6 or more) were aligned to LIS-1 and its 5′ and 3′ flanking sequences using Bowtie (Langmead et al., 2009). The prediction of secondary structure for potential miRNAs and flanking sequences was subsequently performed in LIS-1 and its flanks using mfold (Zuker, 2003).

### Expression analysis of FLAX miRNAs

Expression analysis of miRNAs was performed as described in Melnikova et al. (2014a) for sequence reads generated from the N, NPK, and P libraries. Expression levels of miRNAs in flax libraries were normalized to obtain reads per million (RPM).

The comparison of the expression level of each miRNA in NPK, P, and N libraries was performed using the following formula:

$$\begin{array}{l} fold\;change\;=\;log_{2}\left(RPM\;in\;NPK\;or\;P\;/\;RPM\;in\;N\right) \end{array}$$

*P-*values were obtained according to the calculations of Audic and Claverie (Audic and Claverie, 1997).

### Quantitative real-time PCR (qPCR) analysis

QPCR analysis was performed to evaluate the expression of miRNAs and their predicted targets on extended sampling of 33 flax plants of cultivars “TOST”, “Synichka”, “Antey”, and “Stormont Cirrus” grown under N (14 plants), NPK (seven plants), and P (12 plants) conditions. For six conserved miRNA families (miR168, miR169, miR395, miR398, miR399, and miR408) and 8 novel potential miRNAs (lus-miR-N1, lus-miR-N3, lus-miR-N12, lus-miR-48, lus-miR-246, lus-miR-N306, lus-miR-308, and lus-miR-316) stem-loop primers for reverse transcription (RT) were designed as described previously (Kramer, 2011). The primer sequences are listed in S2 Table.

QPCR for mRNAs, eukaryotic translation initiation factor 3 subunit E (*ETIF3E*), eukaryotic translation initiation factor 3 subunit H (*ETIF3H*), glyceraldehyde-3-phosphate dehydrogenase (*GAPDH*), elongation factor 1-α (*EF1A*), ubiquitin-activating enzyme E1 (*E1*), and ubiquitin conjugating enzyme E2 (*E2*) was performed using a 7500 Real-Time PCR System (Applied Biosystems, USA). The reaction mix (20 μl) contained 1 × 2-FRT PCR mix (Amplisens, Russia), 250 nM of dNTPs mix (Fermentas, Lithuania), 350 nM of forward and reverse primers, 2 U of TaqF polymerase (Amplisens, Russia), 1 × EvaGreen (Biotium, USA), and cDNA. The following program was used for amplification: 95°C for 15 min; 50 cycles of 95°C for 15 s, 61°C for 60 s.

QPCR for miRNAs was performed using the 7500 Real-Time PCR System in a 20 μl reaction mix containing 1 × 2-FRT PCR mix (Amplisens, Russia), 250 nM of dNTPs mix, 1500 nM of forward primer and 700 nM of reverse primer, 2 U of TaqF polymerase (Amplisens, Russia), 1 × Eva Green dye and RT product using the following program: 95°C for 15 min; 50 cycles of 95°C for 15 s, 62°C for 60 s.

Each qPCR reaction was repeated three times. The primer sequences are listed in S2 Table. Sizes of amplification products were validated by electrophoresis in agarose gel.

*EF1A, ETIF3E, ETIF3H, GAPDH* genes (Huis et al., 2010) and lus-miR-N3, lus-miR-N12, lus-miR-N306 were chosen as potential references for qPCR data normalization and tested using BestKeeper (http://www.gene-quantification.de/bestkeeper.html), NormFinder (http://moma.dk/normfinder-software), and geNorm (http://medgen.ugent.be/~jvdesomp/genorm/) software.

Expression level of miRNAs and their target genes was estimated by the following formulas:

$$\begin{array}{l} \Delta \Delta C_{t}^{eff}=\Delta {\left(C_{t}^{eff}\right)}^{normal\;conditions}-\Delta {\left(C_{t}^{eff}\right)}^{stress\;conditions} \end{array}$$

$$\begin{array}{l} \Delta \left(C_{t}^{eff}\right)={\left(C_{t}^{eff}\right)}^{target\;transcript}-{\left(C_{t}^{eff}\right)}^{reference\;transcript} \end{array}$$

$$\begin{array}{l} C_{t}^{eff}=C_{t}\times log_{2}\left(1+E\right) \end{array}$$

where *C*_*t*_, replicate-averaged threshold cycle; *E*, efficiency of reaction for each pair of primers. A higher $\Delta C_{t}^{eff}$ value reflects a lower expression level. All reaction efficiencies were more than 95%. All calculations performed using the program ATG (Analysis of Transcription of Genes; Krasnov et al., 2011; Senchenko et al., 2011), which was compatible with Relative Quantification (RQ) software (Applied Biosystems, USA). The ATG tool allows a full cycle of qPCR data analysis with only well information and fluorescence signal on each PCR cycle used as input data. Main features of the software are as follows: combination of several experiments from different PCR runs in one analysis; computational efficiency evaluation with 3 different methods; *C*_*t*_ estimation; $\Delta C_{t}^{eff}$ and $\Delta \Delta C_{t}^{eff}$ calculation with respective error bars taking into account reaction efficiencies, *C*_*t*_ standard deviations and several reference genes simultaneously; the use of individual (or averaged) control samples for each test specimen; statistical analysis of final data.

The following parameters were determined to study miRNA and mRNA expression: the median $\Delta C_{t}^{eff}$ value (the 50th percentile), i.e., the value dividing the distribution such that 50% values were below and 50% were above it; the range containing 50% of all the values (between the 25th and 75th percentiles); and also the maximum and minimum $\Delta C_{t}^{eff}$ values.

## Results

### Detection of LIS-1 in flax plants

The presence of LIS-1 in the DNA extracted from upper leaves of 33 flax plants (6-week-old) was examined by PCR analysis and agarose gel electrophoresis. LIS-1 was identified in one plant each of “Stormont Cirrus” and “Synichka” grown under P condition. All plants of “Antey” cultivar grown under P or N conditions had LIS-1 while it was absent in all the plants of the “TOST” cultivar grown under any of the N, P, or NPK conditions (S1 Figure).

### Analysis of sRNA sequences in *L. usitatissimum*

Three sRNA libraries from “Stormont Cirrus” plants grown under N, P, and NPK conditions were generated and sequenced. Of the three chosen plants, LIS-1 was absent in those grown under N or NPK conditions, whereas it was present in the one grown under P conditions. A total of 7.2, 11.6, and 7.6 million raw reads were obtained from the deep sequencing of sRNAs for N, P, and NPK conditions, respectively. All the sequences were deposited in the European Nucleotide Archive (ENA) under accession number: PRJEB9528.

**Figure 1:**
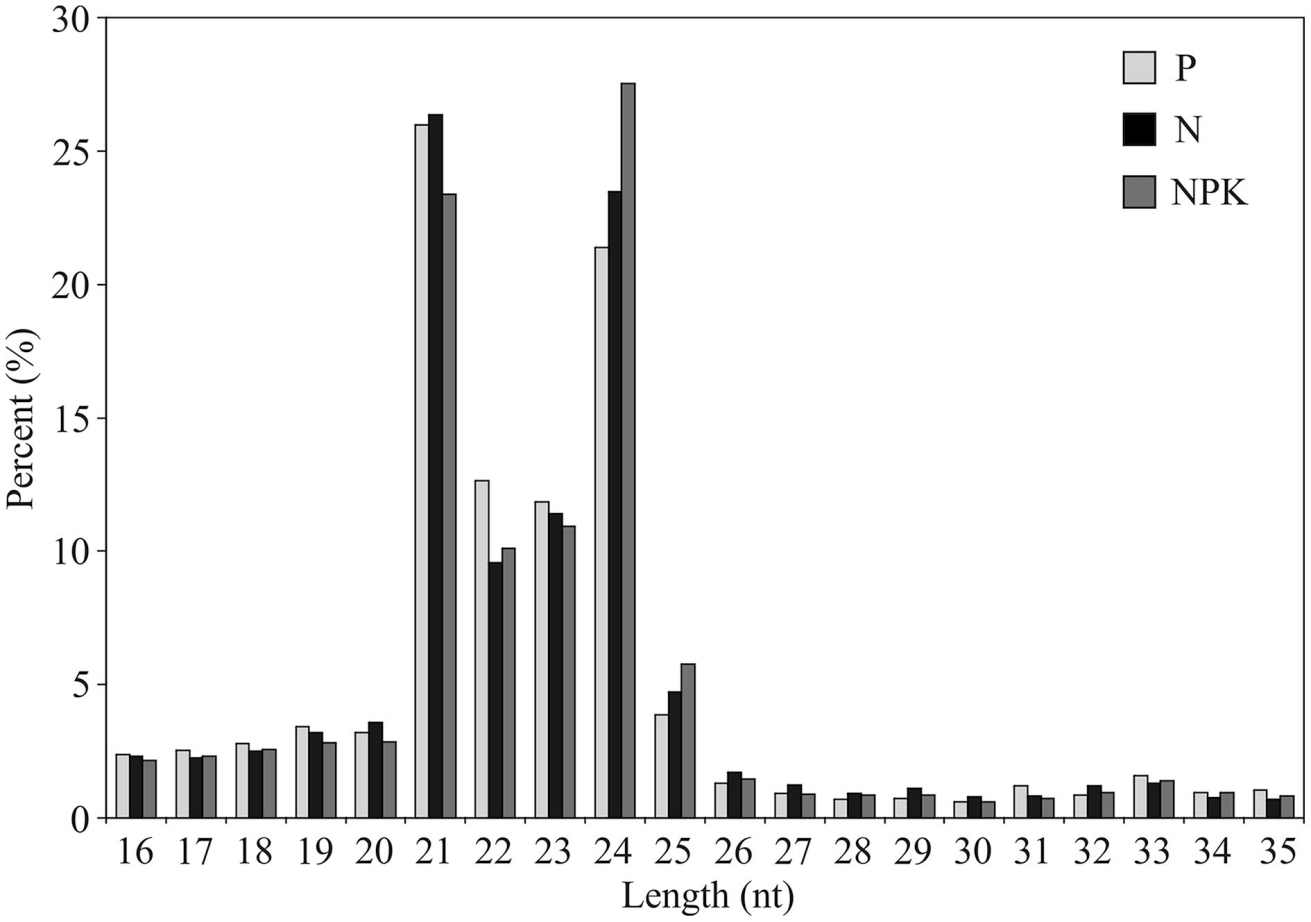
**Length distribution of sRNAs**.

Most of the sRNA sequences obtained from the three libraries were 21–24 nt long, with 21 nucleotide sRNAs (23–26%) and 24 nucleotide sRNAs (21–28%) being the most abundant (Figure 1). The frequency of 24-nt reads in NPK library was higher than 21-nt reads, while in N and P libraries 21-nt reads were the most abundant (Figure 1). The frequency of 24-nt sequences in NPK library was slightly higher than that in N and P libraries. Our results indicate that expression of 24-nt sRNAs could be induced in flax under NPK conditions.

Cleaned reads with abundance 6 or more were mapped to the genome of *L. usitatissimum* (Wang et al., 2012). Most of the reads (77% from P library, 77% from N library, and 78% from NPK library) had at least one match in the flax genome.

To exclude known non-miRNA sRNA sequences from the read dataset, Rfam software was used. The results are presented in Table 1.

**Table 1:** **Annotation of reads from flax small RNA libraries**.

**Feature**	**P**	**N**	**NPK**
Unique sequences with abundance 6 or more	198,269	101,713	142,892
Rfam 11.0	11,626 (5.86%)	4811 (4.73%)	5984 (4.19%)
5S rRNA	739 (0.37%)	350 (0.34%)	427 (0.30%)
snoRNA	1643 (0.83%)	998 (0.98%)	992 (0.69%)
tRNA	1313 (0.66%)	721 (0.71%)	902 (0.63%)

*The data are represented in the format, X (Y), where, X is the number of reads and Y is the percentage of reads in unique sequences with abundance 6 or more*.

### Conserved miRNAs in flax

We identified 96 conserved miRNA homologs from 21 distinct miRNA families in flax. Twelve of these were identified for the first time (S3 Table). The largest miRNA family in flax was miR165/166, which included 20 members. Other families in order of the number of included families were miR396 with nine members, miR319, miR167, and miR156/157 with eight members each, and miR171 and miR172 with six members each. Interestingly, our study revealed many members belonging to the miR165/166 family in flax, which is not the case in other plant species (miRBase, PMRD).

### Novel miRNAs and their targets

High-quality filtered reads (abundance six or more) were mapped to the genome of *L. usitatissimum* and 475 novel potential flax miRNAs with predicted hairpin secondary structure were identified (S4 Table).

For better understanding the functions of these miRNAs, their targets were predicted on the basis of *L. usitatissimum* Unigene Library (http://urgi.versailles.inra.fr/content/download/1198/9515/file/LIN-Unigenes.FASTA.zip) and Joint Genome Institute genomic project, Phytozome v7.0, internal number 200 (ftp://ftp.jgi-psf.org/pub/compgen/phytozome/v8.0/Lusitatissimum/annotation/Lusitatissimum_200_transcript.fa.gz) using a psRNATarget server (http://plantgrn.noble.org/psRNATarget/; Dai and Zhao, 2011; S5, S6 Tables).

**Figure 2:**
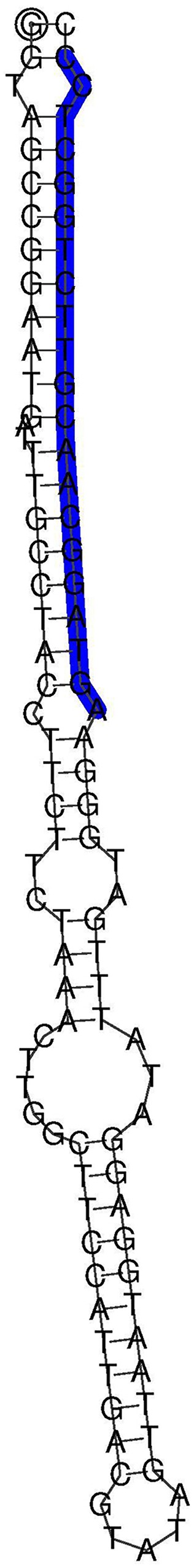
**Predicted secondary structure for lus-miR-N1**.

One of the identified novel potential flax miRNAs was lus-miR-N1 having the sequence 5′-AGUAGGCAA CGUUCUGGCUCC-3′ (Figure 2). The predicted target of this miRNA is the gene encoding ubiquitin-activating enzyme E1 (E1), which catalyzes the first step of the ubiquitination reaction. The regulation of *E1* gene by lus-miR-N1 could probably regulate phosphate starvation response in flax.

### No LIS-1-derived miRNAs were identified

The search for LIS-1-derived miRNAs in the P (LIS-1 present), N (LIS-1 absent), and NPK (LIS-1 absent) sRNA libraries was performed using Bowtie software for NGS sequencing data. Twelve matched LIS-1 sequences (abundance six or more) were identified for P library, eight for N library, and seven for NPK library (S7 Table). Nine of the 12 P library sequences matched the sequences from N and/or NPK libraries, which indicated that these sequences were not derived from LIS-1. Furthermore, the abundance of these sequences in P, N, and NPK libraries was similar. Moreover, we identified three NGS sequences that matched the 3′ flanking sequence of LIS-1 in P sRNA library but these sequences also matched the sequences from N or NPK libraries and had similar abundance. Sequences matching the 5′ flanking sequence of LIS-1 were not found. Therefore, only three unique matched LIS-1 sequences were identified for the P sRNA library. To determine the potential miRNAs in the identified LIS-1 or its 3′ flank matched sequences we used the mfold tool. However, the typical stem-loop structures characteristic of pre-miRNA were not established in any of these sequences signifying the absence of any LIS-1-derived miRNAs in flax.

### miRNA expression analysis on the basis of high-throughput sequencing data

From the 21 identified miRNA families the highest read abundance was detected for miR165/166 family (159 kRPM for P library, 145 kRPM for N library, and 129 kRPM for NPK library). Different members of the same miRNA family had a markedly diverse abundance. For example, in the miR166 family, lus-miR166a expression was 10-20-times higher than the expression of other members of this family in all analyzed libraries. To identify differentially expressed miRNAs in flax, we calculated fold-change values. In previous studies, we identified conserved miRNA families with differential expression under P or NPK conditions (Melnikova et al., 2014a, 2015). In the present work, we report, for the first time, the evaluation of alterations in expression of 475 novel potential miRNAs of flax line “Stormont Cirrus” under P and NPK conditions. Differential expression was revealed for lus-miR-N1 and other novel potential miRNAs (S4 Table).

### Reference gene selection for qPCR data analysis

The selection of candidate reference genes and miRNAs for qPCR evaluation of the alterations in expression levels in flax grown under Pi deficiency or excessive fertilizers was performed. Potential references for qPCR data normalization were chosen on the basis of literature review [*EF1A, ETIF3E, ETIF3H*, and *GAPDH* genes (Huis et al., 2010)] and our NGS and qPCR results (novel potential miRNAs: lus-miR-N3, lus-miR-N12, and lus-miR-N306).

**Figure 3:**
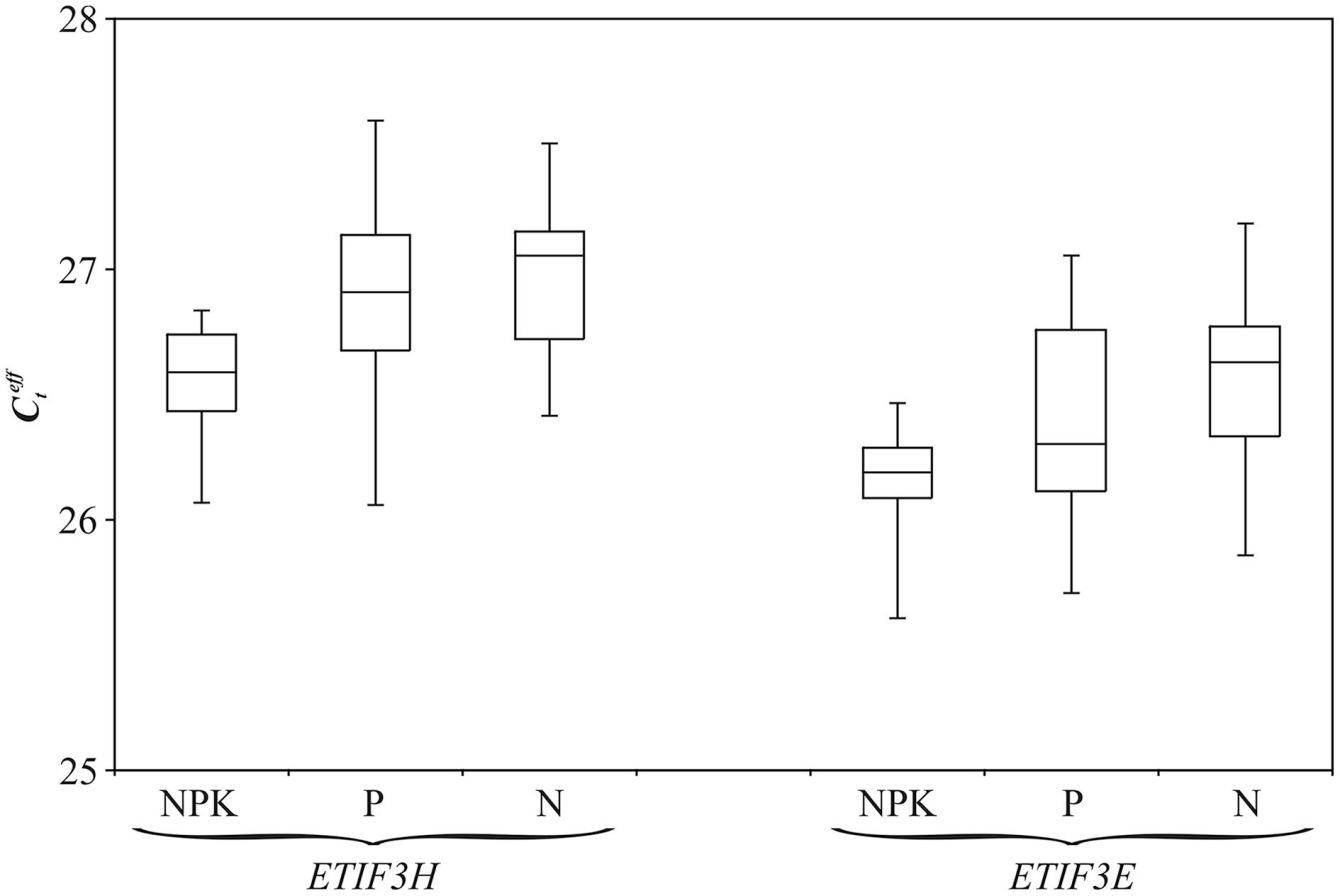
**Distribution of $C_{t}^{eff}$ values for *ETIF3H* and *ETIF3E* genes under excess nutrients (NPK), Pi deficiency (P), and normal (N) conditions**. Rectangles correspond to the range containing 50% of the values (between the 25th and 75th percentiles); the horizontal line inside the rectangle is the median value (the 50th percentile); the bars are the maximum and minimum $C_{t}^{eff}$ values.

BestKeeper, NormFinder, and geNorm software was used to choose the most stably expressed genes/miRNAs in flax grown under different nutrient conditions. The potential references were ranked from the most stable to the least (Table 2). BestKeeper and NormFinder identified *ETIF3H* as the most stable gene, while geNorm chose both *ETIF3E* and *ETIF3H*. Expression of candidate reference miRNAs was less stable than expression of the candidate genes. We therefore selected *ETIF3E* and *ETIF3H* genes for qPCR data normalization in flax grown under imbalanced nutrient conditions. Box plots of the expression level of these genes in the 33 plants are represented in Figure 3.

**Table 2:** **The results of BestKeeper, NormFinder, and geNorm analysis**.

**Rank**	**BestKeeper**	**NormFinder**	**geNorm**
1	*ETIF3H*	*ETIF3H*	*ETIF3E, ETIF3H*
2	*ETIF3E*	*ETIF3E*	
3	*GAPDH*	*EF1A*	*GAPDH*
4	*EF1A*	lus-miR-N12	*EF1A*
5	lus-miR-N3	lus-miR-N3	lus-miR-N3
6	lus-miR-N12	lus-miR-N306	lus-miR-N12
7	lus-miR-N306	*GAPDH*	lus-miR-N306

*Candidate references are ranked from the most stable to the least*.

### miRNA expression analysis using qPCR

**Figure 4:**
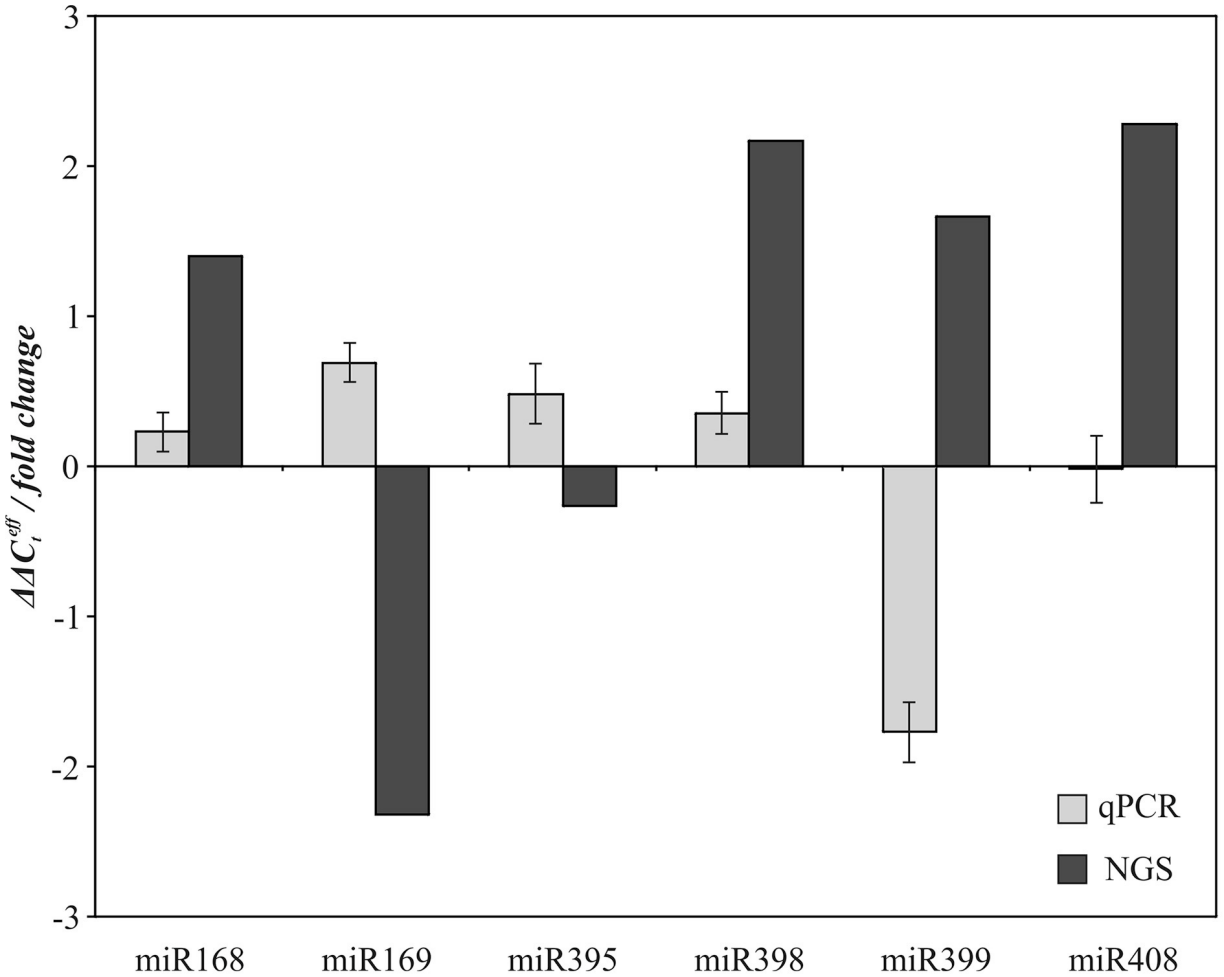
**Comparison of NGS and qPCR data on expression alteration of miR168, miR169, miR395, miR398, miR399, and miR408 in P compared to N conditions**.

Expression of six conserved miRNA families (miR168, miR169, miR395, miR398, miR399, and miR408), which can participate in stress response and nutrient metabolism according to our NGS data, was evaluated using qPCR. The same samples of “Stormont Cirrus” that were used to prepare N and P sRNA libraries for NGS were used for this analysis. The alterations in expression as revealed by NGS were not confirmed by qPCR (Figure 4). Although NGS is suitable for preliminary target selection, it is not considered reliable for quantitative analysis (Shiroguchi et al., 2012). Thus, our high-throughput sequencing data was more suitable for identification of miRNAs than for evaluation of their expression.

**Figure 5:**
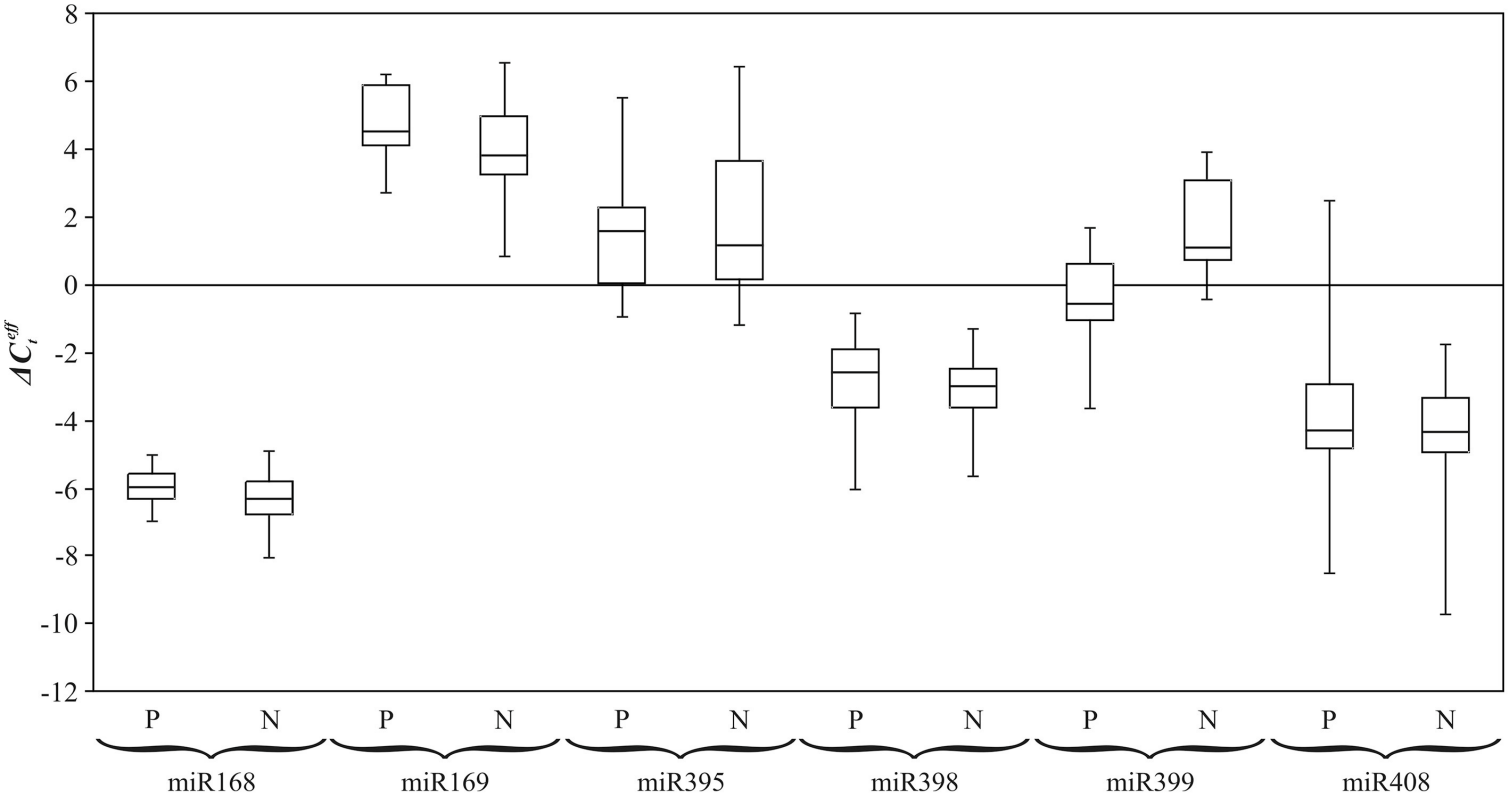
**Expression level ($\Delta C_{t}^{eff}$) of flax miRNA families under Pi deficiency (P) and normal (N) conditions**. QPCR data. Rectangles correspond to the ranges containing 50% of the values (between the 25th and 75th quartiles); the horizontal line inside the rectangle is the median value (the 50th percentile); the bars are the maximum and minimum $\Delta C_{t}^{eff}$ values.

To obtain statistically significant results we assessed the expression level of examined miRNAs on the extended sampling. Previously, we evaluated expression alterations of conserved miRNAs under excess nutrients using qPCR (Melnikova et al., 2015). In the present work, for identification of Pi dependent conserved miRNAs, expression of miR168, miR169, miR395, miR398, miR399, and miR408 was evaluated in 24 flax plants of plastic cultivars “TOST”, “Synichka”, “Antey”, and “Stormont Cirrus” grown under N (14 plants) or P (12 plants) conditions (Figure 5). Up-regulation of miR399 under Pi deficiency was observed: the median of $\Delta C_{t}^{eff}$ value was statistically less for plants grown under deficiency conditions compared to those grown under the N condition (−0.4 vs. 1.4, *p* = 0.004, Mann–Whitney rank-sum test). Thus, we revealed differential expression of miR399 under Pi deficiency.

For miR168, miR395, miR398, and miR408, median of $\Delta C_{t}^{eff}$ for P and N conditions were relatively close: −2.6 and −3.0; −3.9 and −3.9; −5.7 and −6.0; 1.5 and 1.0, respectively (Figure 5). For miR169, the median of $\Delta C_{t}^{eff}$ was 4.6 under P and 4.0 under N conditions. However, for the five miRNA families (miR168, miR169, miR398, miR399, and miR408) expression level alterations under Pi deficiency were not statistically significant.

**Figure 6:**
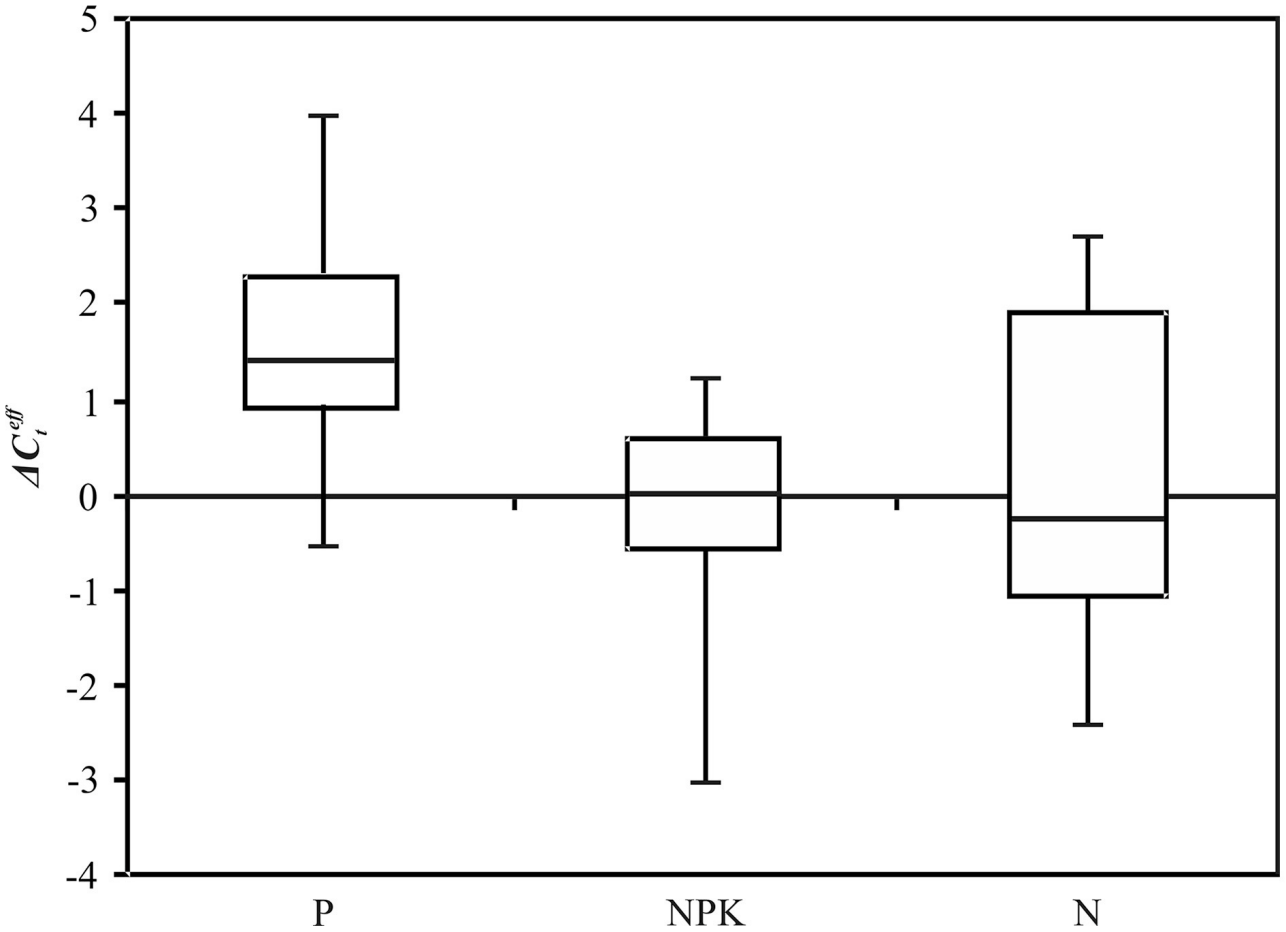
**Expression level ($\Delta C_{t}^{eff}$) of lus-miR-N1 under Pi deficiency (P), excess nutrients (NPK), and normal (N) conditions**. QPCR data. Rectangles correspond to the ranges containing 50% of the values (between the 25th and 75th quartiles); the horizontal line inside the rectangle is the median value (the 50th percentile); the bars are the maximum and minimum $\Delta C_{t}^{eff}$ values.

We further carried out expression analysis of five novel potential miRNAs (lus-miR-N1, lus-miR-48, lus-miR-N246, lus-miR-N308, and lus-miR-N316) on the extended sampling of flax plants under P and NPK conditions. These miRNAs were observed to be differentially expressed on the basis of NGS data and their predicted targets were presumed to play a role in genotroph formation. The satisfactory qPCR results (the efficiency of PCR was higher than 95% with only one specific PCR product and no amplification observed in negative controls of PCR and reverse transcription) were obtained only for lus-miR-N1. Expression of lus-miR-N1 was evaluated in 33 flax plants of four plastic cultivars grown under N, NPK and P conditions (Figure 6). For lus-miR-N1, the median of $\Delta C_{t}^{eff}$ was 1.4 under P, −0.3 under N, and 0.0 under NPK conditions. The alterations in expression level were statistically significant under Pi deficiency compared with excessive fertilizer (*p* = 0.01, Mann–Whitney rank-sum test) and normal conditions (*p* = 0.05, Mann–Whitney rank-sum test). Thus, lus-miR-N1 was down-regulated under Pi deficiency.

Interesting evidence concerning cultivar-specific miRNA expression under Pi deficiency was observed. For miR168, miR398, miR399, and miR408 the expression data were similar for “TOST”, “Synichka”, and “Antey” cultivars. However, for miR169, miR395 and lus-miR-N1 there was a difference in expression alterations within cultivars. Median of $\Delta C_{t}^{eff}$ for miR169 was 4.3 under P and 4.2 under N in “TOST”; 5.8 under P and 2.8 under N in “Antey”; 5.5 under P and 4.0 under N in ‘Synichka’. The median of $\Delta C_{t}^{eff}$ for miR395 was 1.3 under P and 3.8 under N in “TOST”; 2.5 under P and 1.2 under N in “Antey”; 2.6 under P and −0.1 under N in “Synichka.” The median of $\Delta C_{t}^{eff}$ for lus-miR-N1 was 1.4 under P and 0.9 under N in “TOST”; 2.8 under P and −0.7 under N in “Antey”; 1.1 under P and −1.0 under N in “Synichka.”This cultivar specificity of miRNA expression needs further validation.

### Expression of miRNAs and their predicted targets

QPCR analysis revealed two miRNAs with statistically significant expression alterations under different nutrient conditions: conserved miR399 and novel potential lus-miR-N1. We evaluated the mRNA level of their predicted targets in the same samples and performed expression correlation analysis. The negative correlation between miR399 expression and expression of its predicted target gene encoding ubiquitin-conjugating enzyme E2 (E2) was observed (Spearman's rank correlation coefficient *r*_*s*_ = −0.64, *p* < 0.001). A similar result was obtained for lus-miR-N1 and its predicted target gene encoding ubiquitin-activating enzyme E1 (E1) (Spearman's rank correlation coefficient *r*_*s*_ = −0.32, *p* = 0.09). E1 and E2 enzymes participate in ubiquitination: E1 catalyzes the first step and E2 catalyzes the second step of the ubiquitination reaction (Streich and Lima, 2014). Given differential expression of miR399 and lus-miR-N1 under nutrient stress, these data suggest an involvement of the two miRNAs in the phosphate starvation response in flax via regulation of ubiquitination.

## Discussion

### Flax genotroph sRNA sequencing

Plant miRNAs play an important role in regulation of many processes (Sunkar et al., 2007; Guleria et al., 2011; Khraiwesh et al., 2012). In the present study, we sequenced N, P, and NPK sRNA libraries of flax cultivar “Stormont Cirrus”, which is plastic and can undergo heritable phenotypic and genomic changes in response to specific nutrient conditions and form genotrophs (Durrant, 1962). LIS-1 is an effective molecular marker for identification of the S genotrophs (Chen et al., 2005). We tested plant DNA of “Stormont Cirrus” for LIS-1 presence. For sRNA deep sequencing, we chose the plant with LIS-1 from P conditions and the plants without LIS-1 from N and NPK conditions.

The highest read abundance in flax was found for 21- and 24-nt sRNAs. In the NPK library 24-nt reads were the most abundant, but in N and P libraries 21-nt reads had the highest frequency. 21-nt miRNAs and 21-nt trans-acting siRNAs participate in the post-transcriptional gene silencing via mRNA degradation or translation repression (Carthew and Sontheimer, 2009). 24-nt siRNAs are involved in DNA and histone modifications (Hamilton et al., 2002) and 24-nt miRNAs are implicated in DNA methylation (Wu et al., 2010). In our study, an increase in 24-nt sRNA abundance was observed in flax grown under the excessive fertilizer. We suggest that in L genotrophs (formed under NPK conditions), DNA and histone modification and/or methylation processes are more active compared to S genotrophs (formed under P conditions) and plastic flax lines grown under normal conditions.

### Conserved miRNA families in flax

Several studies dedicated to the identification of conserved miRNAs in flax have already been performed. Neutelings et al. (2012) performed a computational homology search of ESTs that identified 20 conserved miRNAs from 13 families in flax. Bioinformatics prediction of candidate miRNAs from the completed flax genome and EST database could identify 12 conserved miRNAs from seven families (Moss and Cullis, 2012). Another computational approach led to the deciphering of 116 conserved miRNAs from 23 families in the flax genome (Barvkar et al., 2013). NGS sequencing of sRNAs of flax plants grown under excess fertilizer allowed us to identify 84 conserved miRNAs (Melnikova et al., 2015). In the present study on sRNAs, we identified 96 conserved miRNA homologs from 21 distinct miRNA families that were expressed in flax plants (S3 Table); 12 of these were revealed in flax for the first time (these miRNAs are highlighted in bold in S3 Table). Thus, our results not only experimentally confirmed the data obtained by computational approach, but also complemented them.

In contrast to the bioinformatics study of Barvkar et al. (2013), in which the maximum number of members was predicted for the miR169 family, our study revealed miR165/166 family to be the most prevalent with 20 members. In other plant species, distribution of the number of members within miRNA families is distinct from those observed in flax (Table 3). In *Arabidopsis thaliana*, miR165/166 family includes only nine miRNAs, while in *Vitis vinifera, Populus trichocarpa, Oryza sativa*, and *Zea mays* the number of members is 8, 17, 14, and 21, respectively. In flax, miR165/166 family had at least two-times more members than other miRNA families. In other plants, the abundance of members in the miR165/166 family is either medium (as in *A. thaliana* and *V. vinifera*) or high, but not the highest (as in *P. trichocarpa, O. sativa*, and *Z. mays*; miRBase 19.0).

**Table 3:** **Number of miRNA family members for *L. usitatissimum* and other plant species according to miRBase 19.0**.

**miRNA family**	Linum usitatissimum^*^	***Arabidopsis thaliana***	***Vitis vinifera***	***Populus trichocarpa***	***Oryza sativa***	***Zea mays***
miR156/157	8	19	9	11	18	20
miR159	3	3	3	6	7	16
miR160	3	3	5	8	8	10
miR162	3	2	1	3	3	3
miR164	2	3	4	6	6	8
miR165/166	20	9	8	17	14	21
miR167	8	5	5	8	13	21
miR168	4	3	1	2	5	4
miR169	1	15	25	32	17	21
miR170/171	6	9	9	14	13	20
miR172	6	7	4	9	0	8
miR319	8	3	5	9	2	6
miR390	2	2	1	4	1	1
miR393	4	2	2	4	3	8
miR394	1	2	3	4	1	2
miR395	2	6	14	10	24	26
miR396	9	2	4	7	13	14
miR397	1	2	1	3	4	2
miR398	3	3	3	3	2	3
miR399	1	6	9	12	11	12
miR408	1	1	1	1	1	1

* *number of miRNA family members for L. usitatissimum is according to the present study data*.

Our data demonstrated that the miR165/166 family also had the highest read abundance, which was more than 70-times higher than in other relatively abundant miRNA families identified in flax. Neutelings et al. (2012) suggested that this family participated in DNA binding, stress response, metabolism, and probably plays a role in tissue identity. One of the potential targets of miR165/166 in flax is UDP-glucuronic acid 4-epimerase gene (Neutelings et al., 2012) that participates in synthesis of plant polysaccharides (Gu et al., 2009). MiRNA165/166 also regulates the expression of HD-ZIP III (class III homeodomain leucine zipper) transcription factors (Kim et al., 2005). In *Arabidopsis*, the restricted expression of HD-ZIP III transcription factors is required for the correct specification of the shoot and root apical meristems (Zhu et al., 2011; Miyashima et al., 2013). MiR166/165 specifically interacts with Argonaute 10 (AGO10) that plays an important role in maintenance of undifferentiated stem cells in the shoot apical meristem and in establishing leaf polarity (Zhu et al., 2011). It can be speculated that the miR165/166 family is actively involved in the regulation of gene expression in flax.

### miRNAs and LIS-1 sequence

Other authors have previously described that the LIS-1 sequence contains several computationally predicted putative miRNAs (Bickel et al., 2012). The search for miRNAs derived from LIS-1 or its 5′ and 3′ flanking sequences was performed using our NGS data for P, N, and NPK flax sRNA libraries. We observed sequences that matched to LIS-1 and its 3′ flanking sequence (S7 Table), but could not identify miRNA-specific stem-loop structures. Moreover, the majority of sequences that matched to LIS-1 were found not only in the flax sample with LIS-1 (P library), but also in the samples without LIS-1 (N and NPK libraries) with similar abundance, indicating that those sequences were not encoded in the LIS-1 region. Therefore, we did not identify miRNAs derived from LIS-1 using our NGS data. LIS-1 possibly contains other non-coding RNAs, the search for which was not the aim of the present work.

### Identification of novel potential miRNA in flax

Although we did not identify any miRNA encoded in the LIS-1 region, a total of 475 novel potential miRNAs and their probable targets were computationally predicted in flax (S4–S6 Tables). The target genes are involved in the majority of processes that occur in flax plants, including cell differentiation, immune response, phosphate homeostasis, hormonal response, plant growth, and development.

NGS allowed us to obtain sequences of potential miRNAs that can be used in further investigations of expression regulation of corresponding genes by other methods, for example qPCR. Differentially expressed under P and NPK conditions, these novel potential miRNAs could participate in genotroph formation and would be worthy of research investigations on extended sampling.

### Expression alterations under P or NPK conditions

To assess miRNA expression on extended sampling and to evaluate the correlation of expression of miRNAs and their predicted targets, we performed a qPCR analysis. To obtain precise and reproducible qPCR results, reliable reference genes are needed. The use of inappropriate reference genes could lead to misinterpretation of expression data. Selection of candidate genes for expression normalization was performed by Huis et al. for different flax plant tissues (Huis et al., 2010). For stem tissues *ETIF3H* was the most stable gene and *GAPDH* was the second based on the of NormFinder analysis. However, there was no data for gene stability in flax under stress conditions. In the present work, expression stability evaluation of potential reference genes and miRNAs was performed for flax grown under Pi deficiency and excessive fertilizer. Four genes (*EF1A, ETIF3E, ETIF3H*, and *GAPDH*) were chosen from the work of Huis et al. (2010). Further, we selected three miRNAs, lus-miR-N3, lus-miR-N12, and lus-miR-N306, on the basis of our high-throughput sequencing data. QPCR was performed for 33 flax plants of cultivars “TOST”, “Synichka”, “Antey”, and “Stormont Cirrus” grown under normal, Pi deficiency, and excessive fertilizer conditions. “Stormont Cirrus” is a confirmed plastic line and can form genotrophs under the certain conditions (Durrant, 1962). For cultivars “TOST”, “Synichka”, and “Antey”, we previously identified LIS-1 insertion in some flax plants grown under Pi deficiency conditions (Melnikova et al, unpublished) that indicates the plastic genome of these cultivars. According to the analysis of qPCR data using BestKeeper, NormFinder, and geNorm software, *ETIF3E* and *ETIF3H* had the maximum expression stability for flax grown under imbalanced nutrient conditions and therefore, we chose them as reference genes for further expression analysis of flax genotrophs.

Expression analysis of six conserved miRNAs (miR168, miR169, miR395, miR398, miR399, and miR408) and one novel potential miRNA (lus-miR-N1) was performed for 33 plants of flax plastic cultivars using qPCR. We identified expression alterations of two miRNAs under P condition; down-regulation of lus-miR-N1 and up-regulation of miR399. It is known that miR399 targets gene encoding E2, which plays an important role in phosphate homeostasis (Allen et al., 2005; Aung et al., 2006; Bari et al., 2006; Pant et al., 2008). In the present work, the negative correlation of expression of miR399 and gene encoding E2 was revealed. We predicted the gene encoding E1 as a target for lus-miR-N1 in flax based on the negative correlation of expression of *E1* and the miRNA. However, in the literature, we found no information about miRNA participation in expression regulation of gene encoding E1 in plants. Thus, to the best of our knowledge, lus-miR-N1 is the first miRNA that could regulate expression of the *E1* gene. Interestingly, both E2 and E1 are involved in the ubiquitination reaction. Ubiquitin is a post-translational modification which regulates cell division, growth, communication, and death (Hershko and Ciechanover, 1998). Ubiquitination involves binding of ubiquitin molecules to target proteins by a specific enzymatic cascade involving E1, E2, and ubiquitin ligase E3 (Bonifacino and Weissman, 1998). Ubiquitinated proteins are targets for degradation (Hershko and Ciechanover, 1998; Brzovic et al., 2006; Shang and Taylor, 2011). In plants, ubiquitin controls phytohormone signaling, the circadian clock, DNA damage repair, response to nutrient deficiency, drought, and other aspects (Smalle and Vierstra, 2004). E1 enzyme initiates the ubiquitination by recognizing and activating cognate ubiquitin and ubiquitin-like proteins followed by transfer to the cognate E2 (Streich and Lima, 2014). Thus, miR399 and lus-miR-N1 could regulate ubiquitination via suppression of E1 and E2 gene expression, and therefore might be involved in adaptation to phosphate starvation in flax plastic lines and genotrophs.

Thus, we identified miRNAs, which were expressed in flax plants under normal, phosphate deficiency, and excess fertilizer conditions, predicted their targets and assessed the expression alterations of miRNAs and their targets using NGS and qPCR. NGS was preferable for identification of miRNAs, while qPCR was suitable for evaluation of their expression. We assessed miRNA expression in flax plants grown under the conditions that can lead to genotroph formation, and in this way obtained new information about the processes occurring in genotrophs.

## Conclusions

In summary, we sequenced sRNAs expressed in *L. usitatissimum* under normal, Pi deficiency, and excess fertilizer conditions. High-throughput sequencing data allowed us to identify 96 conserved and 475 novel potential miRNAs in flax. No LIS-1-derived miRNA was found. QPCR revealed down-regulation of lus-miR-N1 and up-regulation of miR399 under Pi deficiency. Moreover, the negative correlation of expression levels of these miRNAs and their predicted targets, the ubiquitin-activating enzyme E1 gene and the ubiquitin-conjugating enzyme E2 gene, respectively, was demonstrated. Characterization of flax miRNAs provides new insights on the post-transcriptional regulation of gene expression as well as on the processes resulting in genotroph formation.

## Author contributions

NM, NB, AVK, AZ, and OM conceived and designed the work; NM, NB, NKo, AVS, AAK, VL, ASS, AFS, NKi, TR, KK, and AA performed the experiments; AD, MB, GK, and NKo analyzed the data; NM and AD drafted the work. All authors revised the work critically for important intellectual content, approved the version to be published, and agreed to be accountable for all aspects of the work in ensuring that questions related to the accuracy or integrity of any part of the work are appropriately investigated and resolved.

## Funding

The work was financially supported by the Russian Foundation for Basic Research (grants 15-04-06198, 13-04-01770, 14-08-01167) and RAS Presidium Program “Biodiversity of natural systems” (subprogram “Gene pools of nature and their conservation”).

### Conflict of interest statement

The authors declare that the research was conducted in the absence of any commercial or financial relationships that could be construed as a potential conflict of interest. The reviewer, SG, declared a shared affiliation, though no other collaboration, with one of the authors, KK, to the handling Editor, who ensured that the process nevertheless met the standards of a fair and objective review.

## Supplementary material

S1 Figure **Assessment of LIS-1 presence in 33 flax plants of cultivars “Stormont Cirrus”, “Synichka”, “Antey”, “TOST” grown under any of the N, P, or NPK conditions**.

S1 Table **Components of nutrition solutions applied to flax plants grown under normal (N), excess (NPK) and phosphate deficient (P) nutrition conditions**.

S2 Table **Primer sequences**.

S3 Table **Conserved miRNAs identified in flax**.

S4 Table **Novel potential flax miRNAs**.

S5 Table **Predicted targets of flax novel potential miRNAs in *L. usitatissimum* Unigene Library**.

S6 Table **Predicted targets of flax novel potential miRNAs in *L. usitatissimum* JGI genomic project, Phytozome, phytozome v7.0, internal number 200**.

S7 Table **Flax NGS sequences matching LIS-1 and its flanks**.
