# Supplementary figures and images for: Identification, Expression Analysis, and Target Prediction of Flax Genotroph MicroRNAs Under Normal and Nutrient Stress Conditions
Source: Front Plant Sci. 2016 Apr 6;7:399. doi: 10.3389/fpls.2016.00399 (PMC4821855; doi:10.3389/fpls.2016.00399)

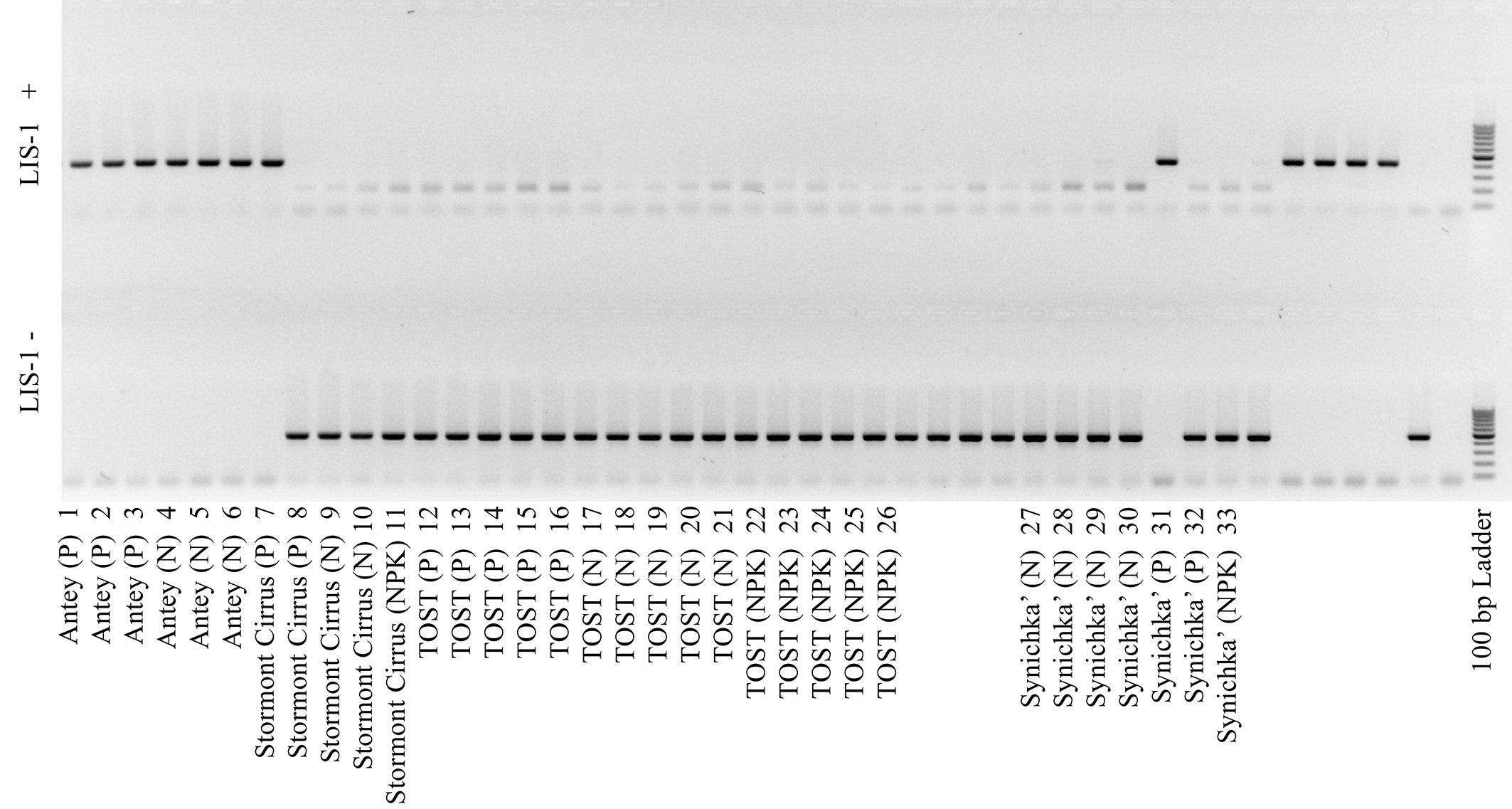

Supplement: S1 Figure — Assessment of LIS-1 presence in 33 flax plants of cultivars “Stormont Cirrus”, “Synichka”, “Antey”, “TOST” grown under any of the N, P, or NPK conditions. [file Image1.TIF]
