# Supplementary material for: Identification, Expression Analysis, and Target Prediction of Flax Genotroph MicroRNAs Under Normal and Nutrient Stress Conditions
Source: Front Plant Sci. 2016 Apr 6;7:399. doi: 10.3389/fpls.2016.00399 (PMC4821855; doi:10.3389/fpls.2016.00399)
Supplement: S1 Table — Components of nutrition solutions applied to flax plants grown under normal (N), excess (NPK) and phosphate deficient (P) nutrition conditions. [file Table1.DOCX]

**S1 Table. Components of nutrition solutions applied to flax plants grown under normal (N), excess (NPK) and phosphate deficient (P) nutrition conditions. 100 ml of nutrition solutions were applied in each pot weekly.**

| **Component** | **N** | **P** | **NPK** |
| --- | --- | --- | --- |
| KNO_3_ | 2.5 mM | 2.5 mM | 2.5 mM |
| Ca(NO_3_)_2_ x 4H_2_O | 2.5 mM | 2.5 mM | 2.5 mM |
| MgSO_4_ x 7H_2_O | 1.0 mM | 1.0 mM | 1.0 mM |
| NH_4_NO_3_ | 0.5 mM | 0.5 mM | 0.5 mM |
| KH_2_PO_4_ | **1.0 mM** | **0.01 mM** | **1.0 mM** |
| H_3_BO_3_ | 25 μM | 25 μM | 25 μM |
| MnCl_2_ x 4H_2_O | 0.5 μM | 0.5 μM | 0.5 μM |
| ZnSO_4_ x 7H_2_O | 0.4 μM | 0.4 μM | 0.4 μM |
| CuSO_4_ x 5H_2_O | 0.25 μM | 0.25 μM | 0.25 μM |
| NaMoO_4_ | 1.25 μM | 1.25 μM | 1.25 μM |
| Fe-EDTA | 45 μM | 45 μM | 45 μM |
| N | - | - | **24 mM** |
| P | - | - | **10 mM** |
| K | - | - | **7 mM** |
| Mg | - | - | **0.3 mM** |
| S | - | - | **0.3 mM** |
| B | - | - | **46 μM** |
| Fe | - | - | **32 μM** |
| Mn | - | - | **13 μM** |
| Zn | - | - | **4 μM** |
| Cu | - | - | **2 μM** |
| Mo | - | - | **0.3 μM** |

*Note*: The differences are shown in bold.
