# Supplementary material for: Identification, Expression Analysis, and Target Prediction of Flax Genotroph MicroRNAs Under Normal and Nutrient Stress Conditions
Source: Front Plant Sci. 2016 Apr 6;7:399. doi: 10.3389/fpls.2016.00399 (PMC4821855; doi:10.3389/fpls.2016.00399)
Supplement: S2 Table — Primer sequences. [file Table2.DOCX]

**S2 Table. Primer sequences.**

| **Primer name** | **Primer sequence** |
| --- | --- |
| miR-R | CCAGTGCAGGGTCCGAGGTA |
| lus-miR169-SL | GTCGTATCCAGTGCAGGGTCCGAGGTATTCGCACTGGATACGACAGGCAA |
| lus-miR169-F | CACGCATAGCCAAGGATGAC |
| lus-miR395-SL | GTCGTATCCAGTGCAGGGTCCGAGGTATTCGCACTGGATACGACGAGTTC |
| us-miR395-F | CACGCACTGAAGTGTTTGGGG |
| lus-miR398-SL | GTCGTATCCAGTGCAGGGTCCGAGGTATTCGCACTGGATACGACCAGGGG |
| lus-miR398-F | CACGCATGTGTTCTCAGGT |
| lus-miR399-SL | GTCGTATCCAGTGCAGGGTCCGAGGTATTCGCACTGGATACGACCAGGGC |
| lus-miR399-F | CACGCATGCCAAAGGAGAGTT |
| lus-miR408-SL | GTCGTATCCAGTGCAGGGTCCGAGGTATTCGCACTGGATACGACGCCAGG |
| lus-miR408-F | CACGCAATGCACTGCCTCTTC |
| lus-miR168-SL | GTCGTATCCAGTGCAGGGTCCGAGGTATTCGCACTGGATACGACTTCCCG |
| lus-miR168-F | CACGCATCGCTTGGTGCAGGT |
| miR-lus-N1-SL | GTCGTATCCAGTGCAGGGTCCGAGGTATTCGCACTGGATACGACGGAGCC |
| miR-lus-N1-F | CACGCAAGTAGGCAACGTT |
| miR-lus-N3-SL | GTCGTATCCAGTGCAGGGTCCGAGGTATTCGCACTGGATACGACGCGATG |
| miR-lus-N3-F | CACGCATTGAATCAGCAGCTC |
| miR-lus-N306-SL | GTCGTATCCAGTGCAGGGTCCGAGGTATTCGCACTGGATACGACGTCTGC |
| miR-lus-N306-F | CACGCATACAGTTTCAGGAACTCT |
| miR-lus-N12-SL | GTCGTATCCAGTGCAGGGTCCGAGGTATTCGCACTGGATACGACCTTCTT |
| miR-lus-N12-R | CACGCAAATGGAGAGTTCGGA |
| lus-miR_N48-SL | GTCGTATCCAGTGCAGGGTCCGAGGTATTCGCACTGGATACGACACACTC |
| lus-miR_N48-R | CACGCAAGAGATGTGAGACAATGA |
| lus-mir_N246-SL | GTCGTATCCAGTGCAGGGTCCGAGGTATTCGCACTGGATACGACACCGTC |
| lus-mir_N246-R | CACGCAAGTCATTCCAACGGTCAG |
| miR-lusN308-SL | GTCGTATCCAGTGCAGGGTCCGAGGTATTCGCACTGGATACGACAGTTGA |
| miR-lusN308-F | CACGCAAGAGAGAATGGATTGAAG |
| miR-lusN316-SL | GTCGTATCCAGTGCAGGGTCCGAGGTATTCGCACTGGATACGACGTGGGC |
| miR-lusN316-F | CACGCACTCGAAATTCTGATGATA |
| ETIF3E-F* | TTACTGTCGCATCCATCAGC |
| ETIF3E-R* | GGAGTTGCGGATGAGGTTTA |
| ETIF3H-F* | CAGCGTGCTTGAAGTAACCA |
| ETIF3H-R* | AACCTCCCTCAAGCATCTCA |
| GAPDH-F* | AGGTTCTTCCCGCTCTCAAT |
| GAPDH-R* | CCTCCTTGATAGCAGCCTTG |
| EF1A-F* | GCTGCCAACTTCACATCTCA |
| EF1A-R* | GATCGCCTGTCAATCTTGGT |
| E1-F | CAAGCCCCCATGTGTACAGT |
| E1-R | CGCAGGAGTCTTCTCAAGCA |
| E2-F | ACTTGGACTCCCAGATTGCC |
| E2-R | CAGAAGCTCCGTGTAACAAACA |

*Note*: SL – steam-loop; R – reverse; F – forward. * Primer sequences are from Huis et al. article [[1](#_ENREF_1)].

**References**

1. Huis R, Hawkins S, Neutelings G. Selection of reference genes for quantitative gene expression normalization in flax (Linum usitatissimum L.). BMC plant biology. 2010;10: 71.
