# Supplementary material for: Identification, Expression Analysis, and Target Prediction of Flax Genotroph MicroRNAs Under Normal and Nutrient Stress Conditions
Source: Front Plant Sci. 2016 Apr 6;7:399. doi: 10.3389/fpls.2016.00399 (PMC4821855; doi:10.3389/fpls.2016.00399)
Supplement: S3 Table — Conserved miRNAs identified in flax. [file Table3.DOCX]

**S3 Table. Conserved miRNAs identified in flax.**

| **miRNA** | **miRNA family** | **Sequence** |
| --- | --- | --- |
| lus-miR156j | miR156/157 | UGACAGAAGAGAGUGAGCACU |
| lus-miR156a | miR156/157 | UGACAGAAGAGAGUGAGCAC |
| lus-miR156k | miR156/157 | UUGACAGAAGAUAGAGAGC |
| lus-miR156l | miR156/157 | UGACAGAAGAGAGUGAGCACA |
| **lus-miR156m** | miR156/157 | **UUGACAGAAGAGAGUGAGCAC** |
| lus-miR156f* | miR156/157 | GCUCUCUAUGCUUCUGUCAUC |
| lus-miR156n | miR156/157 | UGACAGAAGAUAGAGAGCAC |
| lus-miR156b | miR156/157 | UUGACAGAAGAUAGAGAGCAC |
| lus-miR159d | miR159 | CUUGGAUUGAAGGGAGCUCU |
| lus-miR159c | miR159 | UUUGGAUUGAAGGGAGCUCUU |
| lus-miR159e | miR159 | UUUGGAUUGAAGGGAGCUCUA |
| lus-miR160k | miR160 | UGCCUGGCUCCCUGUAUGCC |
| lus-miR160a | miR160 | UGCCUGGCUCCCUGUAUGCCA |
| **lus-miR160e*** | miR160 | **GCGUAUGAGGAGCCAUGCAUA** |
| lus-miR162c | miR162 | UCGAUAAACCUCUGCAUCCA |
| lus-miR162a | miR162 | UCGAUAAACCUCUGCAUCCAG |
| lus-miR162a* | miR162 | GGAGGCAGCGGUUCAUCGAUC |
| lus-miR164a | miR164 | UGGAGAAGCAGGGCACGUGCA |
| **lus-miR164f** | miR164 | **UGGAGAAGCAGGGCACGUGC** |
| lus-miR166j | miR165/166 | UCGGACCAGGCUUCAUCCCC |
| lus-miR166b | miR165/166 | UCGGACCAGGCUUCAUCCCCC |
| lus-miR166k | miR165/166 | UCUCGGACCAGGCUUCAUUC |
| lus-miR166l | miR165/166 | UCUCGGACCAGGCUUCAUUCC |
| lus-miR166m | miR165/166 | CGGACCAGGCUUCAUUCCCC |
| lus-miR166i | miR165/166 | UCGGACCAGGCUUCAUUCCCCC |
| lus-miR166n | miR165/166 | UCGGACCAGGCUUCAUUCCCUU |
| lus-miR166o | miR165/166 | UCGGACCAGGCUUCAUUCCU |
| lus-miR166p | miR165/166 | UCGGACCAGGCUUCAUUCCCU |
| lus-miR166g* | miR165/166 | GGAAUGUUGUCUGGCUCGAGG |
| lus-miR166q | miR165/166 | UCGGACCAGGCUUCAUUCCCG |
| lus-miR166r | miR165/166 | UCGGACCAGGCUUCAUUCCC |
| lus-miR166s | miR165/166 | UCGGACCAGGCUUCAUUCC |
| lus-miR166a | miR165/166 | UCGGACCAGGCUUCAUUCCCC |
| lus-miR166t | miR165/166 | CUCGGACCAGGCUUCAUUCCC |
| lus-miR166f | miR165/166 | UCGGACCAGGCUUCAUUCCUU |
| lus-miR166u | miR165/166 | UCGAACCAGGCUUCAUUCCCC |
| lus-miR166e | miR165/166 | UCGGACCAGGCUUCAUUCCUC |
| **lus-miR166v** | miR165/166 | **UCGGACCAGGCUUCAUUCUC** |
| lus-miR166w | miR165/166 | UCCGGACCAGGCUUCAUUCCC |
| lus-miR167c | miR167 | UGAAGCUGCCAGCAUGAUCUA |
| lus-miR167f | miR167 | UGAAGCUGCCAGCAUGAUCUG |
| lus-miR167j | miR167 | UGAAGCUGCCAGCAUGAUCUGG |
| lus-miR167k | miR167 | UGAAGCUGCCAGCAUGAUCU |
| lus-miR167a | miR167 | UGAAGCUGCCAGCAUGAUCUC |
| lus-miR167b | miR167 | UGAAGCUGCCAGCAUGAUCUU |
| lus-miR167l | miR167 | UGAAGCUGCCAGCAUGAUCUAA |
| **lus-miR167m** | miR167 | **UGAAGCUGCCAGCAUGAUCUGA** |
| lus-miR168b* | miR168 | CCCGCCUUGCAUCAACUGAAU |
| lus-miR168a | miR168 | UCGCUUGGUGCAGGUCGGGAA |
| lus-miR168c | miR168 | UCGCUUGGUGCAGGUCGGGA |
| lus-miR168d | miR168 | UCGCUUGGUGCAGGUCGGG |
| lus-miR169m | miR169 | UAGCCAAGGAUGACUUGCCU |
| lus-miR171b | miR171 | UGAUUGAGCCGUGCCAAUAUC |
| lus-miR171j | miR171 | UGAGCCGAACCAAUAUCACUC |
| lus-miR171k | miR171 | UUGAGCCGUGCCAAUAUCACU |
| lus-miR171i | miR171 | UUGAGCCGUGCCAAUAUCACG |
| lus-miR171l | miR171 | UUGAGCCGCGCCAAUAUCACU |
| **lus-miR171m** | miR171 | **UUGAGCCGUGCCAAUAUCAC** |
| lus-miR172e | miR172 | GGAAUCUUGAUGAUGCUGCAG |
| lus-miR172a | miR172 | AGAAUCUUGAUGAUGCUGCAU |
| lus-miR172e* | miR172 | GGAGCAUCAUCAAGAUUCACA |
| **lus-miR172k** | miR172 | **GUAGCAUCAUCAAGAUUCAC** |
| **lus-miR172l** | miR172 | **GUAGCAUCAUCAAGAUUCACA** |
| **lus-miR172m** | miR172 | **GGAAUCUUGAUGAUGCUGCA** |
| lus-miR319c | miR319 | UUGGACUGAAGGGAGCUCCCA |
| lus-miR319d | miR319 | UUGGACUGAAGGGAGCUCC |
| lus-miR319b | miR319 | UUGGACUGAAGGGAGCUCCC |
| lus-miR319e | miR319 | CUUGGACUGAAGGGAGCUCC |
| lus-miR319a | miR319 | UUGGACUGAAGGGAGCUCCCU |
| lus-miR319f | miR319 | UUGGACUGAAGGGAGCUCCUU |
| lus-miR319g | miR319 | CUUGGACUGAAGGGAGCUCCC |
| lus-miR319h | miR319 | CUUGGACUGAAGGGAGCUCCU |
| lus-miR390a | miR390 | AAGCUCAGGAGGGAUAGCGCC |
| lus-miR390e | miR390 | AGCUCAGGAGGGAUAGCGCC |
| lus-miR393e | miR393 | UCCAAAGGGAUCGCAUUGAUCC |
| lus-miR393f | miR393 | UCCAAAGGGAUCGCAUUGAUCU |
| lus-miR393g | miR393 | UUCCAAAGGGAUCGCAUUGAUC |
| lus-miR393a | miR393 | UCCAAAGGGAUCGCAUUGAUC |
| lus-miR394a | miR394 | UUGGCAUUCUGUCCACCUCC |
| lus-miR395a | miR395 | CUGAAGUGUUUGGGGGAACUC |
| lus-miR395e | miR395 | CUGAAGUGUUUGGAGGAACUC |
| lus-miR396f | miR396 | UUCAAUAAAGCUGUGGGAAG |
| lus-miR396a* | miR396 | GUUCAAUAAAGCUGUGGGAAG |
| lus-miR396a | miR396 | UUCCACAGCUUUCUUGAACUG |
| lus-miR396g | miR396 | GCUCAAGAAAGCUGUGGGAGA |
| lus-miR396h | miR396 | UCCACAGCUUUCUUGAACUG |
| lus-miR396i | miR396 | UUCCACAGCUUUCUUGAACU |
| lus-miR396j | miR396 | CUCAAGAAAGCUGUGGGAGA |
| lus-miR396b | miR396 | UUCCACAGCUUUCUUGAACUU |
| **lus-miR396k** | miR396 | **UUCCACAGCUUUCUUGAACUA** |
| **lus-miR397c** | miR397 | **AUUGAGUGCAGCGUUGAUGA** |
| lus-miR398d | miR398 | UGUGUUCUCAGGUCGCCCCUG |
| lus-miR398e | miR398 | UGUGUUCUCAGGUCACCCCUC |
| **lus-miR398f** | miR398 | **UGUGUUCUCAGGUCACCCCU** |
| lus-miR399h | miR399 | UGCCAAAGGAGAGUUGCCCUG |
| lus-MIR408a | miR408 | AUGCACUGCCUCUUCCCUGGC |

*Note*: miRNAs that were identified in flax for the first time are marked bold.* – miRNA-star.
